# Dr. Joseph L. Howell

**Published:** 1906-08

**Authors:** 


Dr. Joseph L. Howell, of Avoca, N. Y., died at his home July 9, 1906, aged 38 years. His disease was typhoid fever and his death occurred at the end of the second week.

